# Association of infraclavicular axillary vein diameter and collapsibility index with general anesthesia-induced hypotension in elderly patients undergoing gastrointestinal surgery: an observational study

**DOI:** 10.1186/s12871-023-02303-w

**Published:** 2023-10-09

**Authors:** Huijuan Chen, Xianlong Zhang, Lei Wang, Cuijuan Zheng, Shenquan Cai, Wei Cheng

**Affiliations:** 1 Department of Anesthesiology, Affiliated Huaian No.1 Hospital of Nanjing Medical University, 223300 Huaian, Jiangsu China; 2 Department of Anesthesiology, Affiliated Jinling Hospital, Medical School, Nanjing University, 210002 Nanjing, Jiangsu China

**Keywords:** Anesthesia, Hypotension, Infraclavicular axillary vein, Inferior vena cava, Echocardiography

## Abstract

**Background:**

The collapse index of inferior Vena Cava (IVC) and its diameter are important predictive tools for fluid responsiveness in patients, especially critically ones. The collapsibility of infraclavicular axillary vein (AXV) can be used as an alternative to the collapsibility of IVC (IVC-CI) to assess the patient’s blood volume.

**Methods:**

A total of 188 elderly patients aged between 65 and 85 years were recruited for gastrointestinal surgery under general anesthesia. Ultrasound measurements AXV and IVC were performed before induction of general anesthesia. Patients were grouped in accordance to the hypotension after induction. ROC curves were used to analyze the predictive value of ultrasound measurements of AXV and IVC for hypotension after induction of anesthesia. Pearson linear correlation was used to assess the correlation of ultrasound measurements and decrease in mean arterial blood pressure (MAP).

**Results:**

The maximum diameter of AXV(dAXV_max_) and the maximum diameter of IVC (dIVC_max_) were not related to the percentage decrease in MAP; the collapsibility of AXV (AXV-CI) and IVC-CI were positively correlated with MAP changes (correlation coefficients:0.475, 0.577, respectively, *p* < 0.001). The areas under the curve (AUC) was 0.824 (0.759–0.889) for AXV-CI, and 0.874 (0.820–0.928) for IVC-CI. The optimal threshold for AXV-CI was 31.25% (sensitivity 71.7%, specificity 90.1%), while for IVC-CI was 36.60% (sensitivity 85.9%, specificity 79.0%). Hypotension and down-regulation of MAP during induction can be accurately predicted by AXV-Cl after correction for confounding variables.

**Conclusion:**

Infraclavicular axillary vein diameter has no significant correlation with postanesthesia hypotension, whereas AXV-CI may predict postanesthesia hypotension during gastrointestinal surgery of the elderly.

**Trial registration:**

This study was registered in the Clinical Trial Registry of China on 05/06/2022 (ChiCTR2200060596).

**Supplementary Information:**

The online version contains supplementary material available at 10.1186/s12871-023-02303-w.

## Background

Previous studies have shown that general anesthesia induction-related hypotension (GAIH) often occurs during induction of anesthesia for gastrointestinal surgery, with an incidence of about 39.5% [1], and its cause is related to blood volume insufficiency caused by a long period of abstinence from food and drink and gastrointestinal preparation before surgery. At the same time, with the development of medical technology and the continuous acceleration of the population aging, the proportion of elderly gastrointestinal patients in clinical work is increasing. The systemic blood volume of elderly patients decreased with age, and relative volume deficiency exists preoperatively, and prolonged preoperative fasting and abstinence from food and drink for gastrointestinal surgery further lead to preoperative volume deficiency in patients. Hypotension or hypoperfusion during general anesthesia are high risk factors due to the cardiovascular depression and vasodilation or lack of surgical stimulation [2]. In severe cases, this leads to adverse postoperative outcomes such as infarction, cardiac failure, postoperative cognitive dysfunction [3], acute kidney injury, prolonged hospital stay, and threatens to the patient’s life [4, 5]. Therefore, accurate assessment blood volume status in the elderly is critical to predict the occurrence of GAIH in gastrointestinal surgery.

Several studies have shown that the collapsibility index and diameter of the inferior vena cava (IVC) are reliable predictors of fluid responsiveness in patients; and have been shown to have high sensitivity and specificity in predicting the occurrence of hypotension after induction of general anesthesia and after spinal anesthesia [6, 7]. IVC diameters and its collapsibility index (IVC-CI) have been used as practical predictors of blood volume status in the perioperative period.

However, in practice, factors such as obesity, gastrointestinal pneumoperitoneum, abdominal surgery, and surgical sterile area often cause difficulties in measuring IVC [8, 9]. Therefore, there is a need to find another central vein index instead of IVC to assess the blood volume status of elderly patients undergoing gastrointestinal surgery. Zhu et al. [10] showed that the diameter of infraclavicular axillary vein (AXV) was positively correlated with central venous pressure (CVP) in the supine position, and this correlation continued until after fluid resuscitation, and as the patient’s blood volume increased, the diameter of infraclavicular AXV gradually became larger.

There are fewer national and international studies on ultrasound measurement of AXV diameter and its collapse index to assess volume status in elderly gastrointestinal patients to predict postanesthesia hypotension, and there are still no guidelines to follow. The aim of present trial is to investigate the safety of perioperative fluid management by studying the correlation between ultrasound characteristics of the infraclavicular axillary vein and hypotension in elderly gastrointestinal surgery.

## Materials and methods

### Research ethics

This experiment was approved by the hospital ethics committee (KY-2022-049-01, 17 May, 2022) and registered with the China Clinical Trial Registry Centre (ChiCTR2200060596). All patients signed an informed consent form.

### Patients

Between July 2022 and November 2022, 188 elderly patients aged 65–85 years who met ASA 1–3 criteria for general anesthesia for gastrointestinal surgery were recruited at Huai’an No.1 Hospital of Nanjing Medical University. Subjects with taking angiotensin converting enzyme inhibitors (ACEIs) or angiotensin receptor blockers (ARBs), elevated intra-abdominal pressure, severe liver disease, peripheral vascular disease or vascular malformations, pacemaker implantation, autonomic nervous system disorders, signs instability, ejection fraction less than 40%, or unstable angina pectoris were resected.

### Study design

All patients underwent ultrasound measurement of the AXV and IVC before induction of general anesthesia. Patients were divided into the hypotensive and non-hypotensive groups according to whether hypotension occurred after induction. The blood pressure (BP) of patients who were resting preoperatively was the baseline value and was continuously monitored after induction. A decrease in mean arterial blood pressure (MAP) of 20% or more or MAP < 60 mmHg were viewed as hypotension. The incidence of hypotension and the percentage of MAP decrease during the perioperative period were recorded. In the presence of hypotension, an arterial blood sample was taken for blood gas analysis. In the case of hypotension, patients were infused with 300–500 ml of crystalloid or colloidal solution, or given small doses of vasopressors (ephedrine or norepinephrine). We compared measurements in the hypotensive and non-hypotensive groups, including pre-induction AXV and IVC diameter and collapsibility index, hemodynamic indices (MAP, heart rate (HR), CVP, and lactate acid level (Lac)), operation time and intraoperative blood loss, and complications (nausea and vomiting, wound infection and bleeding, deep vein thrombosis, dizziness and headache). ROC curves were used to analyse the predictive value of ultrasound measurements of AXV and IVC for GAIH. Pearson linear correlation analysis was used to assess the correlation of ultrasound measurements and decrease in MAP.

### Ultrasound measurements

**Fig. 1:**
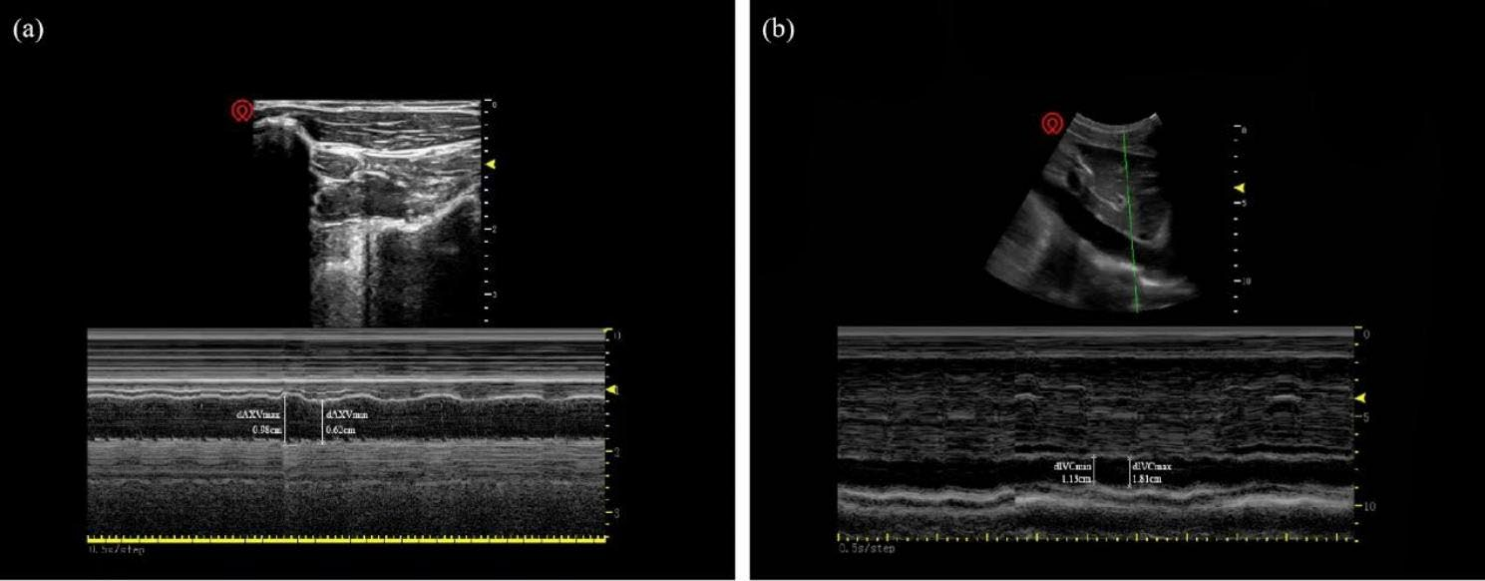
AXV and IVC were measured in M-mode. Measurement of AXV diameter during inspiration (dAXV_min_) and expiration (dAXV_max_) **(a)**. Measurement of IVC diameter during inspiration (dIVC_min_) and expiration (dIVC_max_) **(b)**. (dAXV_max_, maximum diameter of the axillary vein; dAXV_min_, minimum diameter of the axillary vein; dIVC_max_, maximum diameter of the inferior vena cava; dIVC_min_, minimum diameter of the inferior vena cava)

Electrocardiogram (ECG), pulse oximetry, bispectral index (BIS), internal jugular vein and radial artery cannulation were performed upon on admission to the operating room. Each patient was conscious, supine, and breathing spontaneously for at least 5 min prior to the start. All patients underwent ultrasound evaluation of the AXV and IVC. A 3–13 MHz linear transducer (Wisonic Medical Technology Co., Ltd, Shenzhen, China) was used to measure AXV diameter of subjects in the supine position. The probe was scanned from the centre to the outer third of the clavicle for attaining an optimal cross-sectional view of AXV [11] (Fig. 1a). The IVC was visualized using a convex array probe (1–8 MHz). The probe was positioned at the junction of subcostal eminence and the raphe. A cross-sectional image of the IVC can be achieved at 0.5-3 cm from the right atrium which is close to the junction of the hepatic veins [12] (Fig. 1b). We calculated ultrasonic features of AXV and IVC (maximum and minimum diameters, etc.) by M-mode. The collapsibility index can be obtained by the following formula: collapsibility index = (maximum diameter - minimum diameter) / maximum diameter × 100. All measurements were performed by an anaesthesiologist experienced in ultrasonography. Each index was measured three times and the mean was calculated.

### Anesthesia procedure

After ultrasound measurements, the patient was anesthetised using a conventional induction scheme. Midazolam 0.3 mg/kg, etomidate 0.3 mg/kg, sufentanil 0.5 ug/kg, and rocuronium 0.6 mg/kg were administered intravenously sequentially. After loss of reflexes and the muscle relaxation, tracheal intubation and mechanical ventilation were performed. Remifentanil 0.2–0.5 ug/kg/min, propofol 2–6 mg/kg/h, and cisatracurium benzenesulfonate 0.03 mg/kg/h were used to maintain anesthesia by continuous intravenous pumping until the start of surgery. Intravenous anesthesia was combined with 1–2% sevoflurane inhalation when at the start of surgery. The BIS values were maintained in 40–60 range.

### Statistical analysis

#### Sample size

Preliminary results from 38 subjects gave an AUC of 0.65 for the collapsibility index of AXV(AXV-CI). Thus, 150 patients was required (90% power to detect a difference of 0.2 with an AUC of 0.5 to 0.65 using a two-tailed z-test at the 0.05 significance [13]. 188 patients were included allowing for a 20% drop-out rate.

### Data analysis

Normality of data was tested using the Kolmogorov-Smirnov one-sample test. Data are expressed as mean ± standard deviation (SD) for continuous variables and absolute numbers or percentages for categorical variables. Continuous variables were analyzed by independent t-tests after assessment for normality; categorical variables were analyzed using the χ2-test or Fisher’s exact test. Scatter plots were constructed and Pearson correlation coefficient (r) were used to assess the relationship between AXV or IVC results and the downregulations in MAP. ROC curve analyses were used to determine the ability of the maximum diameter of AXV (dAXV_max_) and collapsibility index to predict clinically significant GAIH in all patients. A non-parametric test characterised by Zhang DD was used to compare the two ROC curves [14]. The optimal threshold were determined as the critical value maximising the Youden index (sensitivity + specificity-1) [15]. AUC with 95% confidence intervals was calculated. The association of variables with GAIH was determined by binary logistic regression analysis. Age, American Society of Anesthesiologists (ASA) physical status, pre-existing cerebrovascular disease (CVD), and baseline MAP were adjusted based on literature review and clinical practice. The decrease in MAP from AXV or IVC measurements and other parameters were predicted by multiple linear regression. Independent predictors included age, ASA physical status, pre-existing CVD, baseline MAP, AXV-CI, IVC-CI, dAXV_max_ and maximum diameter of IVC (dIVC_max_). Statistical analyses were performed using IBM SPSS Statistics (version 23.0; IBM Corp., USA) and MedCalc (version 20.0; MedCalc Software, Belgium). Significant *p*-values were set at 0.05.

## Results

The study initially included 188 patients and subsequently excluded 15 patients, of whom 6 patients had poor AXV or IVC visualisation, 3 were on ACEI and ARB medications, 4 were temporarily withdrawnl from the study, and 2 had unexpected airway difficulties. Data were analyzed for the remaining 173 patients. Of all patients analysed, 40 were hypertensive disorders, 12 were on thiazide diuretics, 9 were on calcium channel blockers, 12 were on a beta-blockers, and 7 were not any medication. Types of surgery included gastric cancer (n = 41), colon cancer (n = 58), rectal cancer (n = 47), and appendiceal tumor (n = 27). None of the subjects had a baseline MAP below 60 mmHg. Table 1 summarises the demographic characteristics of the patients.

According to hypotensive criteria, 92 (53.2%) patients presented with GAIH. Of these patients, 76 were treated with rapid infusion to normalise MAP, 10 with norepinephrine and 6 with ephedrine.

**Fig. 2:**
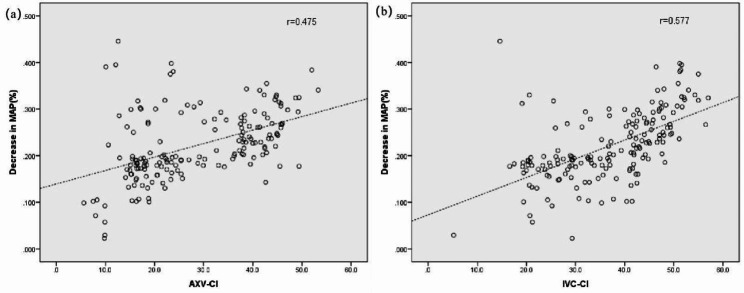
Scatter plots showing AXV-CI and IVC-CI in relation to the decrease in mean arterial blood pressure. (AXV-CI, collapsibility index of the axillary vein; MAP, mean arterial blood pressure; IVC-CI, collapsibility index of the inferior vena cava. Dashed line is trend line)

173 patients were analysed. There was a significant difference in pre-existing CVD between subjects with and without hypotension (*p* = 0.008). However, there were no significant differences in age, sex, body mass index (BMI), ASA physical status, HR, CVP and Lac levels (Tables 2 and 3). MAP decreased after induction compared to before induction in both groups (*p* < 0.001). In addition, the decrease in MAP after induction was greater in the hypotensive subjects than in the non-hypotensive subjects (*p* < 0.001). The dAXV_max_ or dIVC_max_ showed no difference between the hypotensive and non-hypotensive groups (*P* > 0.05); however, AXV-CI (*P* < 0.001) and IVC-CI (*P* < 0.001) were higher in patients with hypotension. The percentage of MAP decrease was positively correlated with AXV-CI (r = 0.475, *p* < 0.001, Fig. 2a) and IVC-CI (r = 0.577, *p* < 0.001, Fig. 2b).

**Fig. 3:**
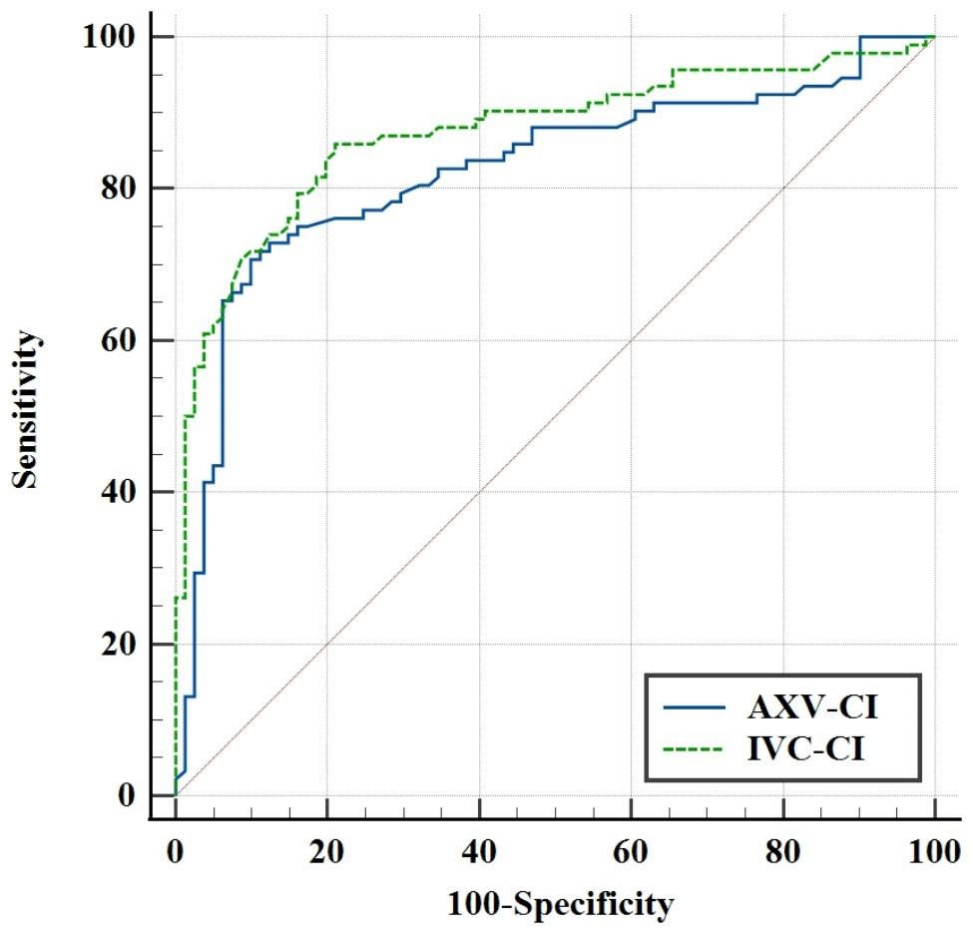
Receiver operating characteristic curves indicates the AXV-CI and IVC-CI for predicting hypotension after anesthesia induction. (AXV-CI, collapsibility index of the axillary vein; IVC-CI, collapsibility index of the inferior vena cava)

As shown in Fig. 3, the ROC curve for GAIH showed high accuracy by AXV-CI, with an AUC of 0.824 (95% confidence interval, 0.759–0.889; *P* < 0.001), and an IVC-CI of 0.874 (95% confidence interval, 0.820–0.928; *P* < 0.001). The optimal cutoff value of AXV-CI was 31.25% with a sensitivity of 71.7% (68.5 − 87.3%) and a specificity of 90.1% (81.5 − 85.6%). The optimal cutoff value of IVC-CI was 36.60% with a sensitivity of 85.9% (77.0 − 92.3%) and a specificity of 79.0% (68.5 − 87.3%). There was no significant difference in the AUC between AXV-CI and IVC-CI (Z = 1.251, *p* = 0.211). It was evident that both AXV-CI and IVC-CI have high predictive value for GAIH in elderly patients undergoing gastrointestinal surgery.

As shown in Table 4, higher AXV-CI, higher IVC-CI, and preexisting CVD were associated with the development of GAIH. After correction for age, ASA physical status, pre-existing CVD, and baseline MAP, AXV-CI (*P* < 0.001) and IVC-CI (*P* < 0.001) were significant independent predictors of GAIH. There was a greater possibility to develop GAIH for patients with a larger collapsibility index, and the odds ratio of AXV-CI was 1.12 (1.07–1.18) while that of IVC-CI was 1.18 (1.11–1.25).

After adjustment for age, ASA physical status, preexisting CVD, and baseline MAP, we found that AXV-CI (*P* < 0.001), IVC-CI (*P* < 0.001), and baseline MAP (*P =* 0.033) were significant predictors of the decrease in MAP after induction of anaesthesia (Table 5).

## Discussion

This prospective trial suggests that preoperative AXV-CI was higher in patients who developed GAIH. AXV-CI was a practical predictor of the occurrence of hypotension and percentage decrease in MAP after correction, as was IVC-CI.

In recent years, the volume status of the patient has received more attention in clinical anesthesia when other factors influencing hypotension cannot be modified, as hypotension is partly dependent on volume status [16–18]. A previous study found [19] that administration of a certain amount of crystals or colloids during induction of anesthesia can help prevent hypotension and reduce total fluid volume. Intravascular volume is an important consideration in maintaining hemodynamics stability and significant decline in cardiovascular function in elderly patients [20–22]. Hypovolemic status is an important factor for GAIH, particularly in gastrointestinal patients. Therefore, accurate assessment of volume status in elderly patients is particularly important in predicting the development of hypotension after anesthesia.

Recently, ultrasound measurements of IVC have become increasingly popular in volume management and fluid therapy [23, 24]. IVC-CI calculated from ultrasound measurements of IVC diameter during spontaneous breathing has been recommended by the American Ultrasound Association as a rapid, non-invasive method of assessing volume status [9]. However, IVC images are usually not well visualised in patients with obesity and gastrointestinal gas collections. In addition, changes in IVC-CI are highly dependent on thoracic and abdominal pressures, which can be influenced by pathophysiological factors such as asthma or respiratory infection.

As a result, some researchers have turned to the infraclavicular AXV, a highly compliant vessel whose volume and hemodynamics vary with humour and respiration. The infraclavicular AXV is located close to the right atrium, and transmural pressure is not affected by intra-abdominal pressure compared to the IVC. In addition, the ultrasound probe compresses the AXV less than other veins (e.g. internal jugular vein), which made the measurements more stable. In 2013, Kent et al. demonstrated that the AXV-CI in the infraclavicular fossa could replace the IVC-CI for assessing the intravascular volume status in critically ill patients, with less measurement bias between the two [11]; furthermore, the time required for imaging and measurement of AXV was shorter than that of the IVC, which was more conducive to timely detection of changes in intravascular volume.

Based on a retrospective analysis of Salmasi and Bijker [25, 26], we defined hypotension as a decrease in MAP of more than 20% from baseline, or below 60 mmHg.

In this study, the incidence of GAIH was 53.2%, which was slightly higher, which may be related to the criteria used for hypotension and the fact that the study population was elderly. AXV-CI and IVC-CI were higher in hypotensive patients. AXV-CI was a significant predictor of hypotension and decrease in MAP (e.g. IVC-CI) after correction.

The AUC for AXV-CI and IVC-CI predicting GAIH were 0.824 and 0.874, respectively, which was close to the previous study on IVC-CI [6], reflecting the good efficacy of AXV-CI and IVC-CI in predicting hypotension. The optimal cut-offs for AXV-CI and IVC-CI were 31.25% and 36.6%, respectively, which were slightly lower than those of the same type of studies. In a study of propofol-induced hypotension by Au et al. [27], patients with IVC-CI ≥ 50% were more likely to have significant hypotension after general anesthesia. This may be related to the type of surgery and the age of the subjects. In addition, it has been shown that a venous collapse index of 30-40% suggests that elderly patients undergoing gastrointestinal surgery are at high risk of GAIH and that anaesthetists can use vasopressors in advance or some volumetric preloading to reduce the incidence of hypotension. Comparative analyses of ROC curve areas between AXV-CI and IVC-CI showed that the differences were not statistically significant, suggesting that AXV-CI can be used as a complementary measure when IVC is difficult to obtain. In the present study, dAXV_max_ and dIVC_max_ were not associated with reduced MAP or CVP. Zhang et al. showed that dIVC_max_ could predict GAIH with an optimal cut-off value of 1.8 cm, which is different from our findings. This may be due to the fact that the diameters of IVC and AXV are susceptible to individual factors such as age, sex, height, weight, and waist circumference making the correlation between BP reduction and diameter parameters insignificant [28]. In addition, previous studies have debated whether maximum diameter can accurately assess intravascular volume. Therefore, we conclude that the collapsibility index is a better indicator of volume status than venous diameter.

There was no statistically significant difference in lactate concentration between the two groups before and after induction, due to fluid resuscitation or the use of vasopressors at the onset of hypotension and the fact that the duration of hypotension was too short to cause changes in the circulation.

There are still some limitations. First, it was a single-center study with a small sample size, that study only included older people. If we had been able to do a multicenter with a large-sample size and subgroup analysis, we might have been able to draw more general conclusions. Second, there are many variables behind hypotension, including cardiac tone, volume, and contractility. Although we excluded patients with poor cardiac function, we cannot be sure that the occurrence of hypotension in patients is fully correlated with volume status. Third, the criteria for hypotension used in this study were different from those used in other studies, which may have led to different results. Further prospective studies are needed to validate and extend the results of this study in different clinical settings.

Overall, this study confirms that AXV-CI is similar to the IVC-CI in predicting the occurrence of hypotension and percentage of MAP fall after induction of anesthesia. Therefore, anesthesiologists may choose this convenient method to initially predict the likelihood of hypotension, which also informs perioperative fluid management.

**Table 1:** Patient demographic data (N = 173)

Variables	
Age (years)	72.25 ± 5.47
Sex (male/female)	98/75
BMI (kg/m^2^)	22.68 ± 2.37
ASA physical status, 1/2/3	14/151/8
Diabetes	17 (9.8)
Hypertension	40 (23.1)
Cardiovascular disease	44 (25.4)
Operation category, cancer of stomach/colon/rectum/appendix	41/58/47/27

Values are mean ± SD or number of patients (% of N). ASA, American Society of Anesthesiologists

**Table 2:** The features of subjects with or without hypotension

	Developed hypotension (n = 173)		
Variables	Yes (n = 92)	No (n = 81)	*P*
Age (years)	72.4 ± 5.2	72.1 ± 5.8	0.748
Sex (male/female)	49/43	49/32	0.338
BMI (kg/m^2^)	22.6 ± 2.3	22.75 ± 2.5	0.691
ASA physical status, 1/2/3	8/79/5	6/72/3	0.814
Diabetes	10 (10.86)	7 (8.64)	0.623
Hypertension	22 (23.91)	18 (22.22)	0.792
Cardiovascular disease	31 (33.7)	13 (16.0)	0.008
Medication			
β-blocker	7 (7.6)	5 (6.2)	0.711
Calcium antagonist	5 (5.4)	4 (4.9)	0.883
Thiazide	6 (6.5)	6 (7.4)	0.819
Operation category			
gastric cancer	23 (25.0)	18 (22.2)	0.668
Colon cancer	32 (34.8)	26 (32.1)	0.709
rectal cancer	25 (27.2)	22 (27.2)	0.998
appendiceal tumors	12 (13.0)	15 (18.5)	0.322
Operation period (min)	167.0 ± 56.4	171.2 ± 56.6	0.620
Amount of bleeding (ml)	118.0 ± 81.0	111.9 ± 7.5	0.588
Nausea and vomiting	6 (6.5)	7 (8.6)	0.598
Postoperative Infection and hemorrhage	1 (1.1)	0 (0)	0.532
Deep vein thrombosis	1 (1.1)	0 (0)	0.532
Dizziness and headache	4 (4.4)	3 (3.7)	0.830

Values are mean ± SD or number of patients (% of N). ASA, American Society of Anesthesiologists

**Table 3:** Comparison of hemodynamics and ultrasound measurements between patients with or without hypotension

	Developed hypotension (n = 173)		
Variables	Yes (n = 92)	No (n = 81)	*P*
MAP_before induction_ (mmHg)	100.38 ± 9.32	97.94 ± 11.92	0.139
MAP _after induction_(mmHg)	72.61 ± 7.61	82.32 ± 10.71	< 0.001
HR_before induction_ (beats/min)	69.32 ± 9.80	69.48 ± 12.26	0.921
HR_after induction_ (beats/min)	62.13 ± 12.06	63.02 ± 12.47	0.633
CVP_before induction_ (mmHg)	6.47 ± 1.69	6.95 ± 2.57	0.152
CVP_after induction_ (mmHg)	6.49 ± 1.82	6.86 ± 2.48	0.264
Lactate_before induction_ (mmol/L)	0.82 ± 0.36	0.78 ± 0.34	0.441
Lactate_after induction_ (mmol/L)	0.84 ± 0.28	0.88 ± 0.40	0.476
dAXV_max_ (cm)	0.86 ± 0.11	0.85 ± 0.11	0.363
dIVC_max_ (cm)	1.88 ± 0.20	1.94 ± 0.22	0.073
AXV-CI (%)	35.06 ± 11.15	20.61 ± 8.41	< 0.001
IVC-CI (%)	43.22 ± 8.54	29.69 ± 8.20	< 0.001

Values are mean ± SD. MAP, mean arterial blood pressure; HR, heart rate; CVP, central venous pressure. dAXV_max_, the maximum diameter of the axillary vein; dIVC_max_, the maximum diameter of the inferior vena cava; AXV-CI, collapsibility index of the axillary vein; IVC-CI, collapsibility index of the inferior vena cava

**Table 4:** Unadjusted and adjusted odds ratios for predicting hypotension after anaesthesia induction

	Unadjusted analysis		adjusted analysis	
	OR(95% confidence interval)	*p*	OR(95% confidence interval)	*p*
Age(years)	1.01(0.96–1.07)	0.746	1.01(0.93–1.10)	0.831
ASA physical status				
1	1		1	
2	0.82(0.27–2.49)	0.730	0.40(0.06–2.49)	0.324
3	1.25(0.21–7.41)	0.806	0.37(0.02–7.30)	0.517
Cerebrovascular disease	2.66(1.28–5.54)	0.009	2.08(0.70–6.18)	0.188
Baseline MAP(mmHg)	1.02(0.99–1.05)	0.134	1.03(0.99–1.08)	0.167
dAXV_max_(cm)	3.61(0.23–56.49)	0.361	0.20(0.00-14.30)	0.462
AXV-CI(%)	1.13(1.09–1.18)	< 0.001	1.12(1.07–1.18)	< 0.001
dIVC_max_(cm)	1.41(0.31–6.43)	0.656	0.79(0.09–6.96)	0.831
IVC-CI(%)	1.19(1.13–1.24)	< 0.001	1.18(1.11–1.25)	< 0.001

ASA, American Society of Anesthesiologists; MAP, mean arterial blood pressure; dAXV_max_, the maximum diameter of the axillary vein; dIVC_max_, the maximum diameter of the inferior vena cava; AXV-CI, collapsibility index of the axillary vein; IVC-CI, collapsibility index of the inferior vena cava

**Table 5:** Multivariate linear regression model for prediction of a decrease in mean arterial blood pressure after anaesthesia induction

	β OR(95% confidence interval)	*p*
Age(years)	0.001(-0.001-0.003)	0.267
ASA physical status^a^	-0.023(-0.048-0.001)	0.062
Cerebrovascular disease	0.005(-0.016-0.026)	0.627
Baseline MAP(mmHg)	0.001(0.000-0.002)	0.033
dAXV_max_(cm)	0.036(-0.044-0.116)	0.380
AXV-CI(%)	0.002(0.001–0.002)	< 0.001
dIVC_max_(cm)	0.046(0.002–0.090)	0.138
IVC-CI(%)	0.003(0.002–0.004)	< 0.001

ASA, American Society of Anesthesiologists; MAP, mean arterial blood pressure; dAXV_max_, the maximum diameter of the axillary vein; dIVC_max_, the maximum diameter of the inferior vena cava; AXV-CI, collapsibility index of the axillary vein; IVC-CI, collapsibility index of the inferior vena cava. ^a^ Reference group = ASA physical status 1

### Electronic supplementary material

Below is the link to the electronic supplementary material.

Supplementary Material 1

Supplementary Material 2

## Data Availability

All data for this study can be obtained from the corresponding author, based on reasonable reasons.
