# Supplementary material for: Association of infraclavicular axillary vein diameter and collapsibility index with general anesthesia-induced hypotension in elderly patients undergoing gastrointestinal surgery: an observational study
Source: BMC Anesthesiol. 2023 Oct 9;23:340. doi: 10.1186/s12871-023-02303-w (PMC10561445; doi:10.1186/s12871-023-02303-w)
Supplement: Supplementary file 2 — Supplementary Material 2 [file 12871_2023_2303_MOESM2_ESM.docx]

**A relevant caption to the raw data**

BMI: body mass index.

ASA: American Society of Anesthesiologists classification.

diabetes mellitus: Patients with diabetes are represented by 1, but not by 0.

Hypertension: Patients with hypertension are represented by 1, but not by 0.

ACEI: Patients taking ACEIs (angiotensin converting enzyme inhibitors) are represented by 1, not by 0.

β-blocker: Patients taking β-blockers (beta-blockers) are represented by 1, not by 0.

Calcium antagonist: Patients taking calcium antagonists are represented by 1, not by 0.

Thiazide: Patients taking thiazides are represented by 1, not by 0.

CVD: Patients with CVD (Cerebrovascular disease) are represented by 1, not by 0.

gastric cancer: Patients undergoing surgery of gastric cancer are represented by 1 and not by 0.

colon cancer: Patients undergoing surgery of colon cancer are represented by 1 and not by 0.

rectal cancer: Patients undergoing surgery of rectal cancer are represented by 1 and not by 0.

appendiceal tumor: Patients undergoing surgery of appendiceal tumor are represented by 1 and not by 0.

AXV_max_: the maximum diameter of axillary vein.

AXV_min_: the minimum diameter of axillary vein.

AXV-CI: collapsibility index of the axillary vein.

IVC_max_: the maximum diameter of the inferior vena cava.

IVC_min_: the minimum diameter of the inferior vena cava.

IVC-CI: collapsibility index of the inferior vena cava.

MAP1: Baseline MAP (mean arterial blood pressure)

HR1: Baseline HR (heart rate)

CVP1: Baseline CVP (central venous pressure)

Lac1: Baseline Lac1 (Lactate level)

MAP2:the lowest MAP after anesthesia induction

MAP decrease: the percentage of MAP decrease

Group: Patients with hypotension after anesthesia induction are in group 1, while those without hypotension are in group 2

HR2: the lowest HR (heart rate) after anesthesia induction

CVP2: the lowest CVP (central venous pressure) after anesthesia induction

Lac2:the Lactate level at the time of the lowest MAP

Operation period: Operating period for each patient

Amount of bleeding: Amount of bleeding for each patient

Nausea and vomiting: Patients with postoperative nausea and vomiting are represented by 1 and not by 0.

Postoperative Infection and hemorrhage: Patients with postoperative Infection and hemorrhage are represented by 1 and not by 0.

Deep vein thrombosis: Patients with deep vein thrombosis are represented by 1 and not by 0.

Dizziness and headache: Patients with dizziness and headache are represented by 1 and not by 0.
